# Therapeutic effects of oxaliplatin-based neoadjuvant chemotherapy and chemoradiotherapy in patients with locally advanced rectal cancer: a single-center, retrospective cohort study

**DOI:** 10.1186/s12957-018-1403-9

**Published:** 2018-06-05

**Authors:** Takashi Okuyama, Shinichi Sameshima, Emiko Takeshita, Ryuji Yoshioka, Yukinori Yamagata, Yuko Ono, Nobumi Tagaya, Tamaki Noie, Masatoshi Oya

**Affiliations:** 1 Department of Surgery, Dokkyo Medical University, Koshigaya Hospital, 2-1-50, Minami Koshigaya, Koshigaya, Saitama 343-8555 Japan; 2 Department of Pathology, Dokkyo Medical University, Koshigaya Hospital, 2-1-50, Minami Koshigaya, Koshigaya, Saitama 343-8555 Japan

**Keywords:** Locally advanced rectal cancer, Oxaliplatin-based neoadjuvant chemotherapy, Neoadjuvant chemoradiotherapy, Relapse-free survival

## Abstract

**Background:**

Neoadjuvant chemoradiotherapy (NACRT) has now become the standard treatment for locally advanced rectal cancer (LARC). NACRT has decreased local relapse (LR) rate in patients with LARC; however, distant relapse has recently attracted much attention. This study aimed to assess the feasibility and efficiency of neoadjuvant chemotherapy (NAC) for LARC.

**Methods:**

Data on patients with cT3/4 and N+ rectal cancer who were treated in our institution from April 2010 to February 2016 were reviewed retrospectively. Twenty-seven patients who received 2–9 cycles of oxaliplatin-based NAC and 28 patients who received NACRT (45 Gy delivered in 25 fractions and 5-fluorouracil-based oral chemotherapy) were analyzed. The primary and secondary endpoints of the present study were the 3-year relapse-free survival (RFS) and the local and distant relapse rates, respectively.

**Results:**

Regardless of the kind of neoadjuvant therapy, no patient experienced any grade 3–4 therapy-related adverse events. The frequent toxic events were grade 1 diarrhea in patients with NACRT and neutropenia in patients with NAC. A significantly higher proportion of patients with NAC underwent laparoscopic surgery and anterior resection (*p* = 0.037 and *p* = 0.003, respectively). The percentages of patients with lymph node yield less than 12 in the NAC group, and those in the NACRT group were 26 and 68%, respectively (*p* = 0.002). Comparing the NAC with the NACRT groups, the local relapse and distant relapse rates were 7.4 and 7.1% and 7.4 and 18%, respectively. There were no significant differences in 3-year RFS and 4-year overall survival (OS) between NAC and NACRT (3-year RFS 85.2 vs. 70.4%, *p* = 0.279; 4-year OS 96.3 vs. 89.1%, *p* = 0.145, respectively). With an analysis excluding patients who received postoperative adjuvant chemotherapy, no patients who received NAC had a distant relapse, and there was a significant difference in 3-year RFS compared with the NACRT groups (94.4 vs. 63.2%, *p* = 0.043).

**Conclusion:**

These outcomes suggest that the therapeutic effect of oxaliplatin-based NAC is at least equal to that of NACRT and that NAC is a feasible and promising option for LARC.

## Background

Therapeutic strategies for locally advanced rectal cancer (LARC) have evolved significantly during the last three decades. In Western countries, neoadjuvant chemoradiotherapy (NACRT) followed by total mesorectal excision (TME) is currently the standard treatment option for patients with LARC [1]. However, it is well known that, although NACRT reduces the local recurrence to a rate less than 10%, distant relapse occurs in 25–40% of patients [2–4]. This distant relapse rate is almost equal to the local relapse rate in patients who receive no neoadjuvant therapy (NAT) [5]. In addition, several patients who received NACRT had an unfavorable response, with unnecessary toxicity and a delay in surgery.

Early systemic treatment may be effective to reduce distant relapse and prevent dissemination of micrometastases. Neoadjuvant chemotherapy (NAC) without radiation for LARC has recently been explored to reduce distant relapse and avoid the toxicities of radiation without compromising local control [6–8]. Although a few randomized, controlled studies are ongoing, there is no literature comparing NACRT and NAC performed at a single institution where the surgical technique for LARC is relatively homogeneous [9–11].

In the present study, different neoadjuvant treatment strategies for LARC, short-term survival results, and oncological results at our institution were compared.

## Methods

### Patients and specimens

This study was undertaken with the approval of the Institutional Review Board (IRB) for the protection of human subjects at the Dokkyo Medical University, Koshigaya Hospital.

A total of 55 patients (33 men, 22 women) with LARC treated with or without NAT followed by surgical resection at our institution between April 2010 and February 2016 were included. NAC and NACRT were performed in 27 and 28 patients, respectively. The median follow-up periods for relapse-free survival (RFS) and overall survival (OS) were 41.3 and 45.4 months, respectively. The eligibility criteria included (1) previously untreated, histologically confirmed, locally advanced adenocarcinoma of the rectum with or without nodal involvement and requiring surgery; (2) stage cT3/4 and cN + cM0; (3) World Health Organization (WHO) performance status 0–1; and (4) age < 80 years. Patients with clinical obstruction underwent temporary diverting ostomies.

All patients were initially evaluated by colonoscopy. To obtain an accurate diagnosis and investigate the Kirsten rat sarcoma viral oncogene homolog (KRAS) mutation state, multiple sites (4–7 sites) of the primary tumor were sampled and necrotic tissues were avoided. Pelvic magnetic resonance imaging (MRI) was used to determine the local extent of the disease, including the depth of invasion and lymph node metastases. When it was not possible to precisely determine nodal status on MRI, clinical nodal status was considered node-negative (N-negative) if there were no perirectal and lateral pelvic lymph nodes (LPLNs) with diameter < 7 mm and node-positive (N-positive) if there were one or more perirectal and lateral pelvic lymph nodes with diameter ≥ 7 mm.

On MRI, tumor and lymph node shrinkage rates were defined respectively as T- and N-downstaging, and shrinkage was defined as a decrease in size ≥ 30% in the comparison before and after NAT. Radiologic tumor response was evaluated in accordance with the Response Evaluation Criteria in Solid Tumors guidelines [12]. Chest- and abdominopelvic-computed tomography (CT) were used to diagnose distant metastases. Clinicopathological findings were analyzed based on the TNM classification of malignant tumors, according to the Union for International Cancer Control (UICC) [12]. Histological tumor regression after NAT was graded according to the Japanese Society for Cancer of the Colon and Rectum: grade 0, no regression; grade 1a, minimal effect (necrosis less than one third of the lesion); grade 1b, mild effect (necrosis less than two thirds but one third or more of the lesion); grade 2, moderate effect (necrosis more than two thirds of the lesion); and grade 3, absence of residual tumor cells [13]. For convenience, grades 0, 1a, and 1b were classified as non-responders and grades 2 and 3 were classified as responders.

### Neoadjuvant therapy

The NACRT regimen consisted of external beam radiation (45 Gy delivered in 25 fractions) to the whole pelvis including the pelvic sidewall and 5-fluorouracil (5-FU)-based oral chemotherapy. Patients underwent surgical resection with curative intent 6–8 weeks after the completion of CRT. The NAC regimen was determined by the tumor histology including KRAS mutations. If patients had wild-type KRAS, 2 cycles of SOX: S-1 (80 mg/m^2^) on days 1 to 14, oxaliplatin (85 mg/m^2^) on day 8, plus cetuximab (400 mg/m^2^ as an initial weekly dose, followed by 250 mg/m^2^/week) were given. If patients had a non-wild-type KRAS, 1–2 cycles of 2 cycles of SOX, 2–9 cycles of mFOLFOX6 in 2-week cycles (oxaliplatin, 85 mg/m^2^, l-leucovorin 200 mg/m^2^, 5-fluorouracil bolus 400 and 2400 mg/m^2^ continuous infusion over 46 h), or 2–3 cycles of XELOX, with capecitabine (1000 mg/m^2^) twice daily on days 1–14 and oxaliplatin (130 mg/m^2^) on day 1, were given. Patients underwent surgical resection with curative intent 4–6 weeks after completion of NAC. Toxicity was assessed before each 2–3-week cycle, according to the National Cancer Institute Common Terminology Criteria for Adverse Events, version 4.0.

### Surgery

TME with/without sphincter preservation was to be carried out whenever feasible, according to the standardized techniques, using a laparoscopic or open approach. Laparoscopic and open approaches were performed in 39 (71%) patients and 16 (29%) patients, a sphincter-preserving operation (low anterior resection) was performed in 27 (49%) patients, and an abdominoperineal resection (APR) was performed in 28 (51%) patients, respectively. Creation of a temporary diverting ostomy was depended on the discretion of the primary surgeon. Lateral pelvic lymph node dissection (LPLND) was added if there was suspicious node positivity at the area of the pelvic sidewall based on a pelvic MRI before NAT.

The primary and secondary endpoints of the present study were 3-year RFS and local and distant relapse rates.

### Postoperative adjuvant therapy and follow-up evaluations

Postoperative adjuvant chemotherapy was usually recommended to patients with ypT4 and ypN+ or those with positive circumferential resection margin for a total of − 6 months including the duration of NAC and NACRT using a regimen including 5-FU-leucovorin, capecitabine, SOX, or FOLFOX. However, the selection of PAC of regimen was basically depended on the primary surgeon. Patients had regular follow-up every 3–4 months, including clinical evaluations and physical examinations at least 5 years after surgery. Contrast-enhanced CT scans of the chest/abdomen and pelvis were performed every 6 months, and proctoscopy were performed every 1 year. Local and distant relapse was diagnosed by clinical evaluation and imaging.

### Statistical analysis

Categorical variables are presented as numbers (percentage) and were analyzed with *χ*^2^ and Fisher’s exact tests, and continuous variables are presented as medians (range) and analyzed with the Mann-Whitney *U* test. RFS and OS curves were plotted using the Kaplan-Meier method and compared using the log-rank test. Statistical analysis was performed using SPSS version 23 (IBM Japan Ltd., Tokyo, Japan). A *p* value less than 0.05 was considered significant.

## Results

### Baseline demographic and cancer characteristics

Patients who received NAC and NACRT showed similar demographic characteristics except for operation approach and operation method (Table 1). A significantly higher proportion of patients with NAC underwent laparoscopic surgery and anterior resection (*p* = 0.037 and *p* = 0.003, respectively). There were no significant differences in other clinicopathological characteristics including age, sex, pre-treatment serum CEA level, tumor size, distance from the anal verge, tumor differentiation, clinical-T and clinical-N category, LPLND, and postoperative adjuvant chemotherapy.

**Table 1:** Baseline demographics and cancer characteristics

	Treatment group		
Characteristics	NAC (27)	NACRT (28)	*p* value
Age, median (range), years	66.0 (40–79)	68.0 (42–78)	*p* = 0.64*
Gender (male), *n* (%)	17 (63%)	16 (57%)	*p* = 0.79
Pretreatment serum CEA level, average (range), ml/dl	4.7 (0.9–646)	6.2 (1.5–1244)	*p* = 0.26*
Tumor size, average (range), cm	5.0 (2–10)	4.9 (2–10)	*p* = 0.91*
Distance from anal verge (< 5 cm), *n* (%)	17 (63%)	19 (68%)	*p* = 0.78
Tumor differentiation, *n* (%)			
Well, moderate	26 (96%)	27 (96%)	*p* = 1.00
Muc	1 (3.7%)	1 (3.6%)	
cT category, *n* (%)			
pT3	24 (89%)	22 (71%)	*p* = 0.46
pT4a	3 (11%)	5 (18%)	
cN category, *n* (%)			
pN1	18 (67%)	17 (61%)	*p* = 0.78
pN2	9 (33%)	11 (39%)	
Operation approach (lap), *n* (%)	23 (85%)	16 (57%)	*p* = 0.037
Operative method, *n* (%)			
Low anterior resection	19 (70%)	8 (29%)	*p* = 0.003
Abdominoperineal excision	8 (30%)	20 (71%)	
LPLN dissection (yes), *n* (%)	9 (33%)	5 (18%)	*p* = 0.16
Postoperative adjuvant chemotherapy (yes), *n* (%)	9 (33%)	12 (43%)	*p* = 0.58

*Mann-Whitney *U* analysis

### Comparison of neoadjuvant therapeutic response with clinicopathological findings and toxicities

Surgical site infection (SSI) occurred more frequently in patients with NACRT than in those with NAC (71 vs. 29%, *p* < 0.001). The percentages of patients with lymph node yield (LNY) less than 12 in the NAC group and the NACRT group were 26 and 68%, respectively (*p* = 0.002, Table 2). Except for the abovementioned factors, there were no significant differences in almost all factors, including normalization of CEA values post NAT, RECIST evaluation, pathological grade, distance from anal verge after NAT, circumferential resection margin (CRM), lymphovascular invasion, improved invasion (pT), and lymph node metastasis (pN), pLPLN metastasis, and ypStage.

**Table 2:** The comparison of neoadjuvant therapeutic response to clinicopathological findings

	Treatment group		
Characteristics	NAC (27)	NACRT (28)	*p* value
Median follow-up period of RFS	47.0	32.5	*p* = 0.23*
Median follow-up period of OS	51.0	35.0	*p* = 0.23*
Normalization of CEA value post NAT (yes), *n* (%)	7 (54%)	8 (50%)	*p* = 0.57*
Distance from anal verge after NAT (< 5 cm), *n* (%)	15 (56%)	14 (50%)	*p* = 0.79
Pathological response (responder), *n* (%)	9 (33%)	13 (46%)	*p* = 0.23
*Complete response	1 (3.7%)	5 (18%)	
Circumferential margin (negative), *n* (%)	26 (93%)	26 (93%)	*p* = 0.51
Lymphovascular invasion (present), *n* (%)	15 (56%)	13 (46%)	*p* = 0.34
ypT stage (ypT0–2), *n* (%)	11 (41%)	12 (43%)	*p* = 0.55
ypN (absent), *n* (%)	21 (78%)	21 (75%)	*p* = 0.53
Lymph node yield, *n* (%)			
Less than 12	7 (26%)	19 (68%)	*p* = 0.002
12 or more	20 (74%)	9 (32%)	
pLPLN (present), *n* (%)	1/9 (11%)	0/5	*p* = 0.95
ypStage, *n* (%)			
ypStage0 (CR)	1 (3.7%)	4 (14%)	*p* = 0.59
ypStage1	7 (26%)	6 (21%)	
ypStage2	13 (48%)	12 (43%)	
ypStage3	6 (22%)	6 (22%)	

*Mann-Whitney *U* analysis

Regardless of the kind of NAT, no patient experienced any grade 3–4 therapy-related adverse events. The frequent toxic events were grade 1 diarrhea in patients with NACRT and neutropenia in patients with NAC (Table 3).

**Table 3:** Number and percentage of patients with most common grade 1–2 toxicities by common terminology criteria for adverse events

NAC	Grade	
Category of toxicity	Grade 1, *n* (%)	Grade 2, *n* (%)
Neutropenia	4 (15%)	0
Thrombocytopenia	1 (3.7%)	0
Hepatic dysfunction	1 (3.7%)	0
Neuropathy	2 (7.4%)	0
Dermatopathy	0	1 (3.7%)
Vomiting	1 (3.7%)	0
NACRT	Grade	
Category of toxicity	Grade 1, *n* (%)	Grade 2, *n* (%)
Kidney dysfunction	1 (3.7%)	0
Radiation dermatitis	1 (3.7%)	0
Diarrhea	2 (7.4%)	0

### Comparisons of neoadjuvant therapeutic response with patient outcomes

Eleven of 55 (20%) patients who received NAT developed metastatic disease, with the first confirmed site indicated in Table 4. In the NAC and NACRT groups, 2 patients had local relapse (NAC group 7.4% and CRT group 7.1%). On the other hand, 2 patients who received NAC and 5 patients who received NACRT had distant relapse (NAC group 7.4% and NACRT group 18%). With re-analyses excluding 21 patients who received PAC, no patients who received NAC had a distant relapse (Table 5).

**Table 4:** Summary of postoperative relapses

Location	Treatment group	
	NAC	NACRT
Overall relapse (present), *n* (%)	4 (15%)	7 (25%)
Local relapse (present), *n* (%)	2 (7.4%)	2 (7.1%)
Distant relapse (pulmonary and liver), *n* (%)	2 (7.4%)	5 (18%)

**Table 5:** Summary of postoperative relapses excluding patients who received PAC (*n* = 34)

Location	Treatment group	
	NAC (*n* = 18)	NACRT (*n* = 16)
Overall relapse (present), *n* (%)	1 (5%)	5 (31%)
Local relapse (present), *n* (%)	1 (5%)	2 (13%)
Distal relapse (pulmonary and liver), *n* (%)	0	3 (19%)

**Fig. 1:**
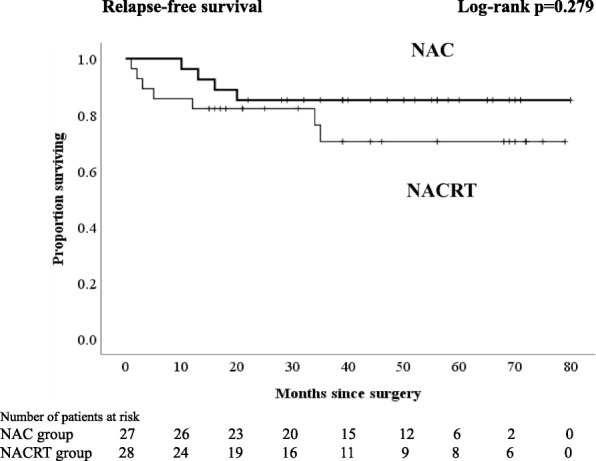
Kaplan-Meier curves for patients who received neoadjuvant chemotherapy or neoadjuvant chemoradiotherapy. Relapse-free survival

**Fig. 2:**
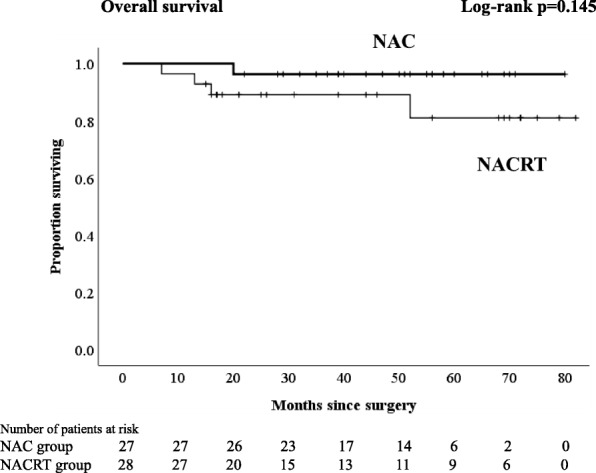
Kaplan-Meier curves for patients who received neoadjuvant chemotherapy or neoadjuvant chemoradiotherapy. Overall survival

**Fig. 3:**
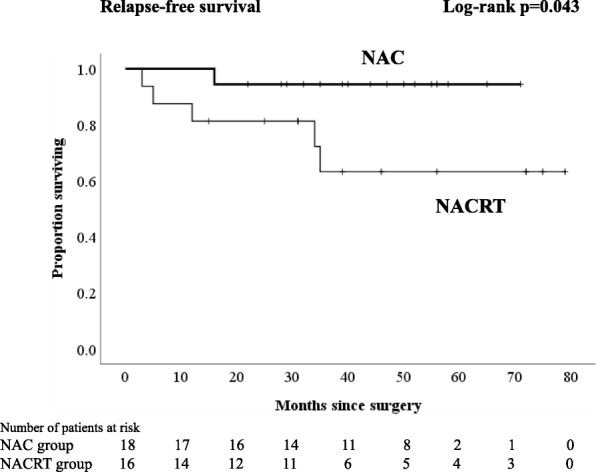
Kaplan-Meier curves for patients who received neoadjuvant chemotherapy or neoadjuvant chemoradiotherapy excluding patients who received PAC. Relapse-free survival

**Fig. 4:**
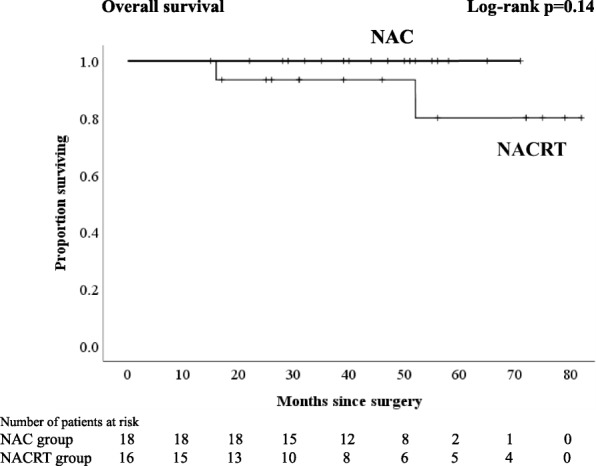
Kaplan-Meier curves for patients who received neoadjuvant chemotherapy or neoadjuvant chemoradiotherapy excluding patients who received PAC. Overall survival

Although there were no significant differences in 3-year RFS and 4-year OS between NAC and NACRT (3-year RFS 85.2 vs. 70.4%, *p* = 0.279, Fig. 1; 4-year OS 96.3 vs. 89.1%, *p* = 0.145, respectively, Fig. 2), in analyses excluding patients who received PAC, there was no significant difference in OS (4-year OS NAC 100% vs. NACRT 93%, *p* = 0.14, Fig. 4), but there was a significant difference in RFS (3-year RFS NAC 94.4% vs. NACRT 63.2%, *p* = 0.043, Fig. 3).

In total, 5 patients (CRT 4 patients, NAC 1 patient) died of metastatic rectal cancer during follow-up (Fig. 4). Six patients with metastatic disease were alive, one of whom received radiotherapy for pelvic lymph node relapse and was cancer-free.

## Discussion

The present study compared the therapeutic effects of NAC and NACRT with regard to clinicopathological findings, therapeutic toxicities, relapses, and survival curves at a single institution. Only one study so far has been published on the comparison of these treatment strategies for LARC patients [14]. In the present study, although the numbers of patients treated either by NAC or NACRT were small, several interesting results were found with regard to perioperative factors. Anal sphincter preservation was more often possible after NAC than after NACRT, even though the distance between the distal margins of the tumor and the anal verge before treatment did not differ between patients treated with NAC and those treated with NACRT. SSI was less frequent after NAC than after NACRT. In addition, the distant relapse rate of patients who received NAC was low compared with that of NACRT (7.4 vs. 18%), and there was a significant difference in 3-year RFS excluding patients who received postoperative adjuvant chemotherapy (NAC vs. NACRT; 94.4 vs. 63.2%, *p* = 0.043).

Local relapse rates did not differ between NAC and NACRT, and they were almost equal to the local relapse rates reported in previous trials [1, 2]. Although local relapse has been the most significant concern in patients after curative resection of LARC, distant relapse has also attracted much attention in recent years [2–4, 15]. Distant relapse was slightly less frequent after NAC than after NACRT. No significant differences in OS and RFS were found between NAC and NACRT.

In analyses excluding patients who received PAC for the purpose of eliminating the effect of PAC, no patients who received NAC had a distant relapse, and consequently, RFS was significantly longer after NAC than after NACRT. These results suggest that NAC may be at least equally useful to NACRT for the prophylaxis of not only local relapse, but also distant relapse in patients with LARC.

After both NAC and NACRT, lymph node metastasis was not found (ypN0) in approximately 75% of patients and an improvement of depth of invasion to ypT0–1 was achieved in approximately 40% of patients after both NAC and NACRT. Pathological lymph node metastasis is the strongest prognostic factor in patients with colorectal cancer. Recent studies have shown a reduction of number of LNY after NACRT because of the reduction of the size and number of lymph nodes (LNs) by NACRT, due to fibrosis or disappearance [16–18]. Furthermore, LNY is an important prognostic factor because a smaller LNY may result in critical failure in identifying metastatic LNs, and several recent studies have also shown that LNY is also an important prognostic indicator in patients who received NACRT [19–21]. Data from a Dutch nationwide trial on rectal cancer showed that 12 or more LNs were found in only 19% of patients who received NACRT [22]. In the present study, on routine pathological examination, the percentages of patients with 12 or more LNs in the NAC group and the NACRT group were 74 and 32% (*p* = 0.002), respectively. These values were less than those in previous studies, especially in the NACRT group [20, 23–25]. Miller et al. reported that the mean LNY after NACRT was decreased by 7 to 53% compared with that without NACRT and that the mean value of the decrease in the studies used in their review was 28% [16]. In the present study, 3 of 14 (21%) patients after NACRT with less than 12 LNs developed relapses. Therefore, the required number of LNs for the precise staging of patients after NACRT needs to be revised by further analyses.

It has been generally acknowledged that NACRT increases the risk of treatment-related toxicities such as gastrointestinal, dermatologic, and neurologic toxicities in patients with LARC [1, 26]. However, there were no grade 3/4 acute treatment-related toxicities in either the NACRT or the NAC group in the present study. Several trials of NACRT suggested that NACRT increased the incidence of surgical morbidity, especially perineal wound healing after APR [27, 28]. In the present study, SSI was significantly more frequent in the NACRT group than in the NAC group (71 vs. 22%, *p* < 0.001). Therefore, neoadjuvant oxaliplatin-based chemotherapy appears to be a safe therapeutic option, and the risk of treatment-related toxicities and surgical complications does not exceed that of NACRT.

Currently, the PROSPECT study, comparing NAC alone and NAC plus NACRT (conventional CRT), is ongoing as a randomized phase II/III trial in North America with participation of all US and Canadian cooperative groups. More recently, the FOWARC study is also being carried out as a randomized phase III trial comparing NAC with or without radiation and NACRT (conventional CRT). Although disease-free survival (DFS) as the endpoint of the FOWARC study is still under evaluation, Deng et al. have concluded that NAC alone showed a lower pCR rate than NAC with radiation, but it had a similar downstaging rate to that of NACRT with less toxicity [9]. We should wait and evaluate their outcomes to improve the treatment cure for patents with LARC.

Studies comparing NAC and NACRT head-to-head for patients with LARC are very few. Cassidy et al. surveyed 21,707 patients who would meet the eligibility criteria of the PROSPECT trial from the National Cancer Data Base (NCDB) and compared NACRT and NAC (neoadjuvant multiagent chemotherapy) for clinical T2N1 (cT2N1), cT3N0, or cT3N1 rectal cancers [14]. Using propensity score-matched analysis to avoid selection bias, they observed a worse OS in the NAC group than in the NACRT group. They concluded that elimination of CRT for patients who have cT2N1, cT3N0, or cT3N1 rectal cancers should be avoided until the results of the PROSPECT trial are published. In Cassidy’s study, however, the number of patients who received NAC alone was much less than that who received NACRT. The regimens and duration of NAC were not described and might have differed by institution and the period when the patients were treated. The patterns of relapse are not specified either. Therefore, the evaluation of NAC and NACRT for patients with LARC requires the common regimen including contents, duration, and postoperative treatment.

To the best of our knowledge, there has been no report from a single institution comparing NAC and NACRT, though the numbers of patients of both the NAC and NACRT groups were too small and the regimens of NAC were not homogeneous in the present study. The selection of either NAC or NACRT was not randomized and was usually based on the patient’s preference. Surgeons’ preference may also influence the selection of either of NAC and NACRT, because the patient’s decision is often influenced by the surgeon’s explanation when obtaining informed consent. In addition, some of the patients refused PAC by themselves despite the surgeons’ recommendation based on the pathological findings. Therefore, no conclusion can be drawn from the present study alone. However, the results suggest that future prospective studies to explore the safety and efficacy of NAC for LARC would be valid.

## Conclusions

In conclusion, the present study suggests that the therapeutic effect of oxaliplatin-based NAC appears equal to, or may be even greater than that of, NACRT in several clinicopathological factors such as reduced toxicity, increased number of LNY, and improved RFS in patients who did not received PAC. Although we are unable to specify the best regimen and duration of NAC from the present study, oxaliplatin-based NAC might be effective for both local and distant control of LARC. Further studies regarding the regimen and duration of NAC is warranted in order to identify the best method of NAC for LARC.

## Abbreviations

APR: Abdominoperineal resection
CEA: Carcinoembryonic antigen
CRM: Circumferential resection margin
CT: Computed tomography
DFS: Disease-free survival
LARC: Locally advanced rectal cancer
LN: Lymph node
LNY: Lymph node yield
LPLND: Lateral pelvic lymph node dissection
MRI: Magnetic resonance imaging
NAC: Neoadjuvant chemotherapy
NACRT: Neoadjuvant chemoradiotherapy
NAT: Neoadjuvant therapy
NCDB: National Cancer Data Base
OS: Overall survival
PAC: Postoperative adjuvant chemotherapy
RECIST: Response Evaluation Criteria in Solid Tumors
RFS: Relapse-free survival
SSI: Surgical site infection
TME: Total mesorectal excision
UICC: Union for International Cancer Control
WHO: World Health Organization
